# Retraction: Qinning *et al.* Predictive Factors and Clinical Outcomes of Stent Malapposition in Calcified Lesions After PCI: A Retrospective Observational Study Based on OCT Assessment. Reviews in Cardiovascular Medicine. 2025; 26: 44024

**DOI:** 10.31083/RCM49405

**Published:** 2025-12-24

**Authors:** Reviews in Cardiovascular Medicine Editorial Office

The Editor-in-Chief has retracted the article entitled “[Predictive Factors and Clinical Outcomes of Stent Malapposition in Calcified Lesions After PCI: A Retrospective Observational Study Based on OCT Assessment]” ([1]) due to significant concerns regarding the reliability and integrity of the data presented.

After publication, the authors identified a significant issue that compromises the reliability and validity of the study’s findings. Specifically:

Fig. 2 contains images that are identical to those in Fig. 3A,C of a previously published paper [2].

These duplications of images raise serious questions about the validity of the results and the adherence to ethical standards of research.

Because this issue cannot be adequately addressed through correction or revision, the authors have voluntarily requested the retraction of the article to uphold academic and scientific integrity. The authors apologize to the editors, reviewers, and readers for any inconvenience caused. The journal has agreed to the retraction. This article is therefore officially retracted.
